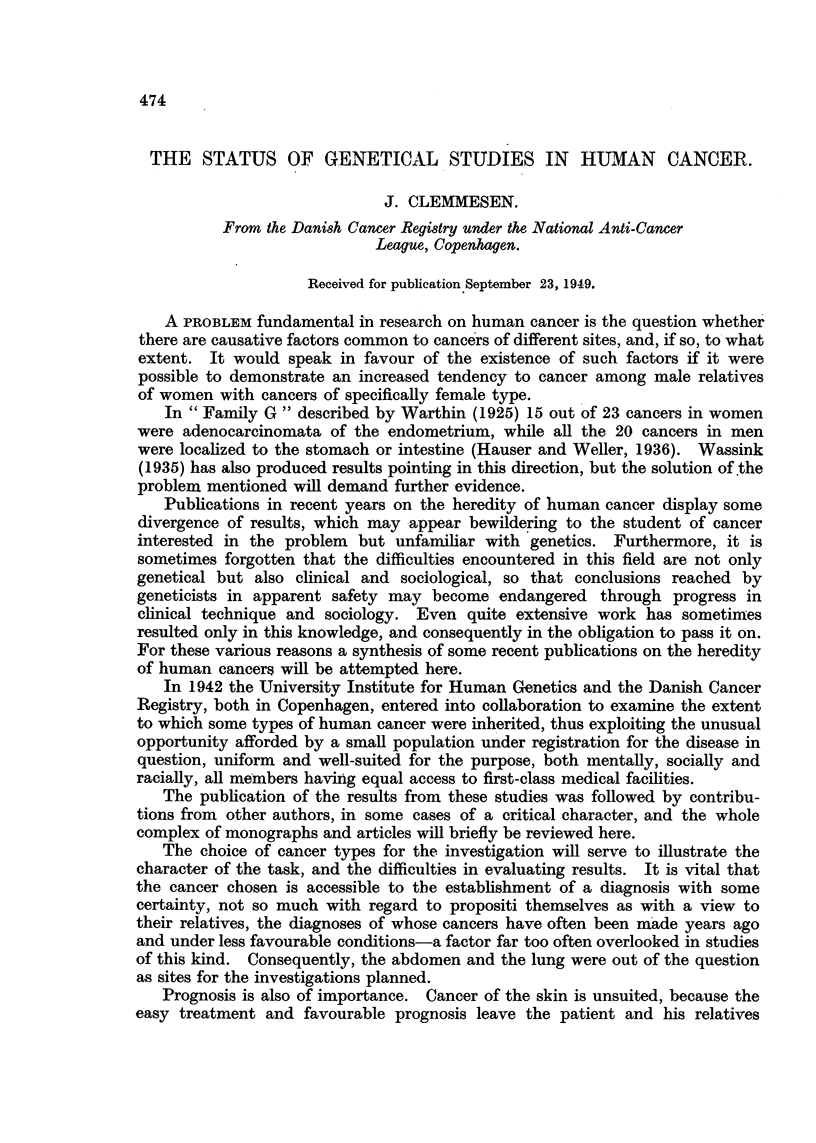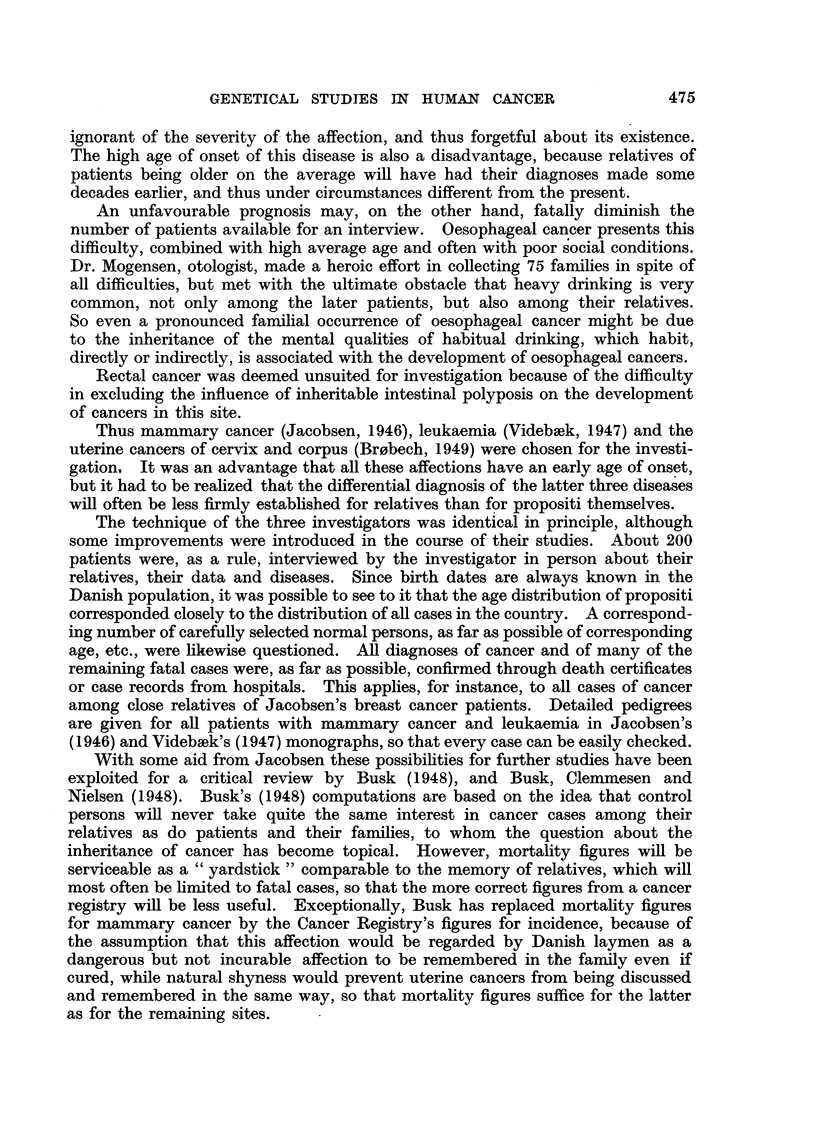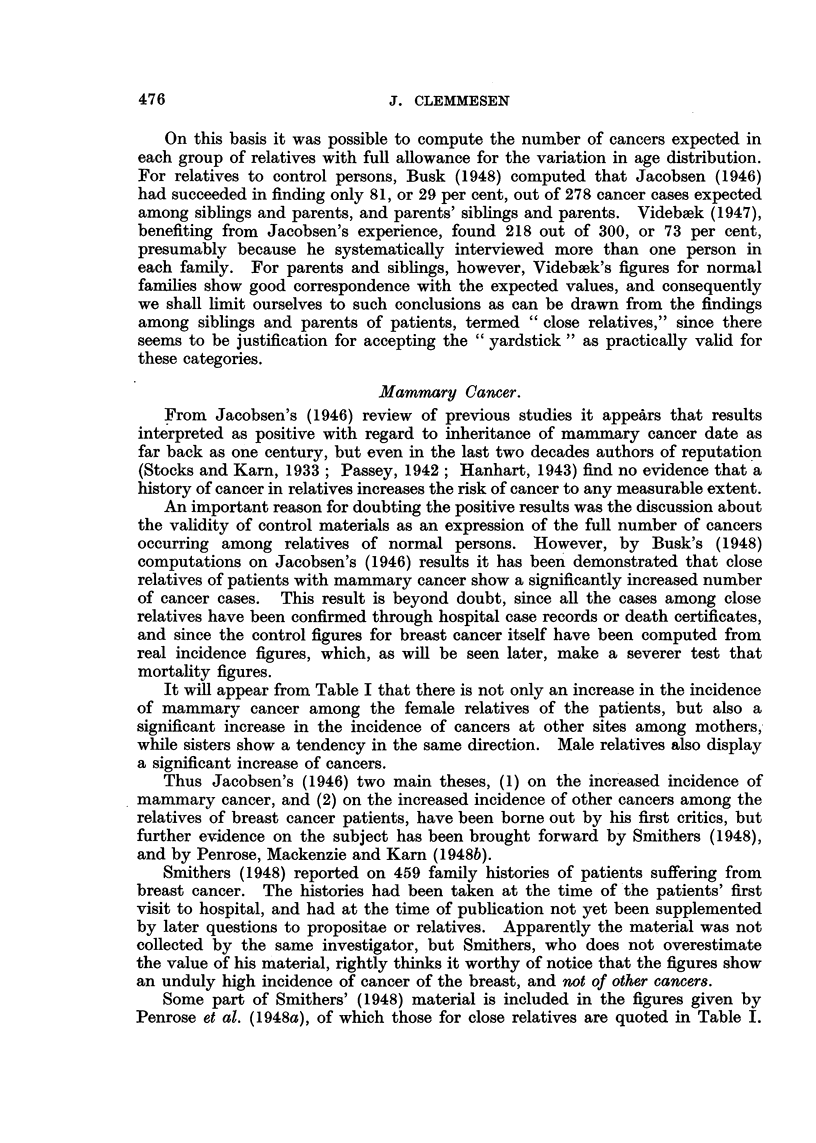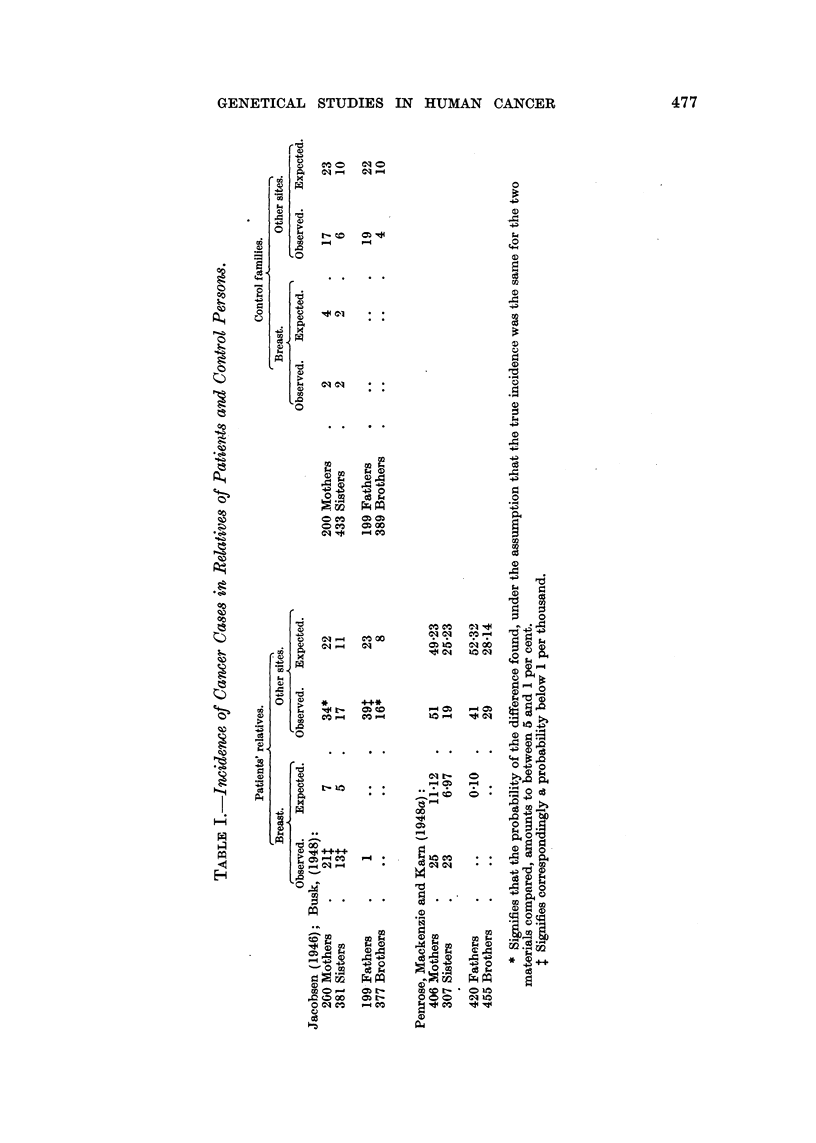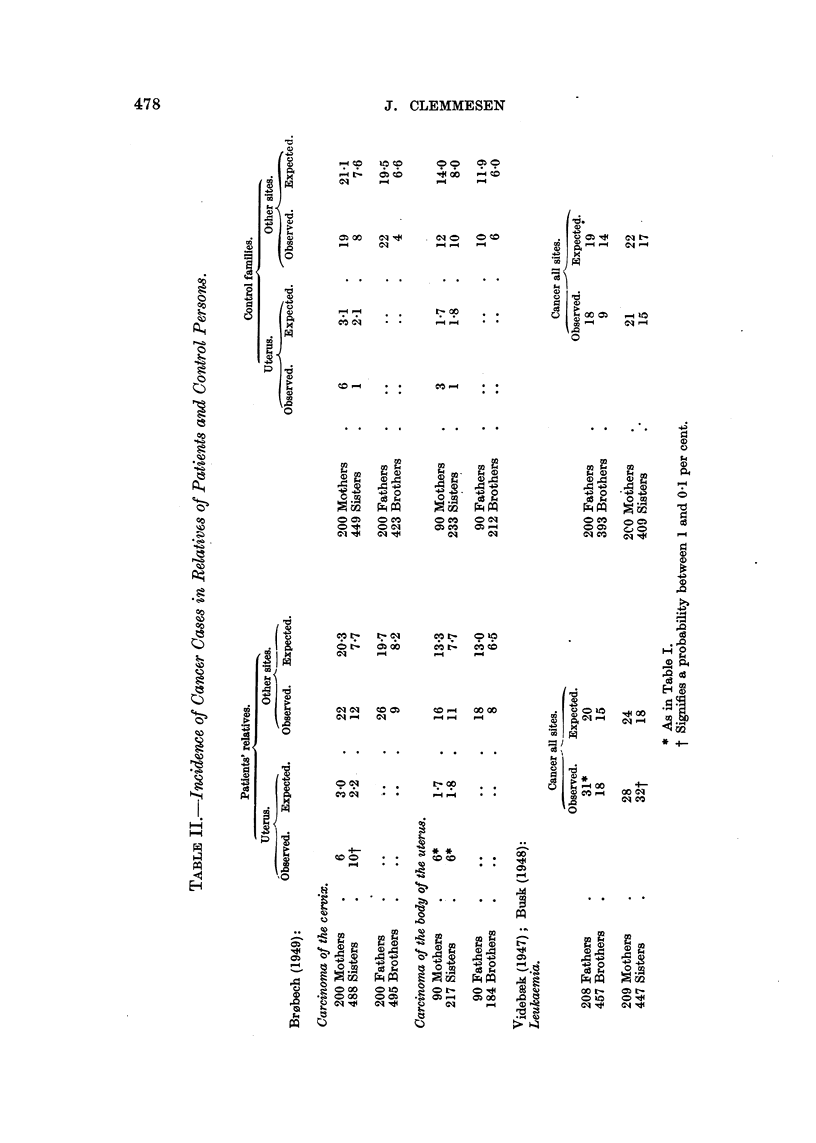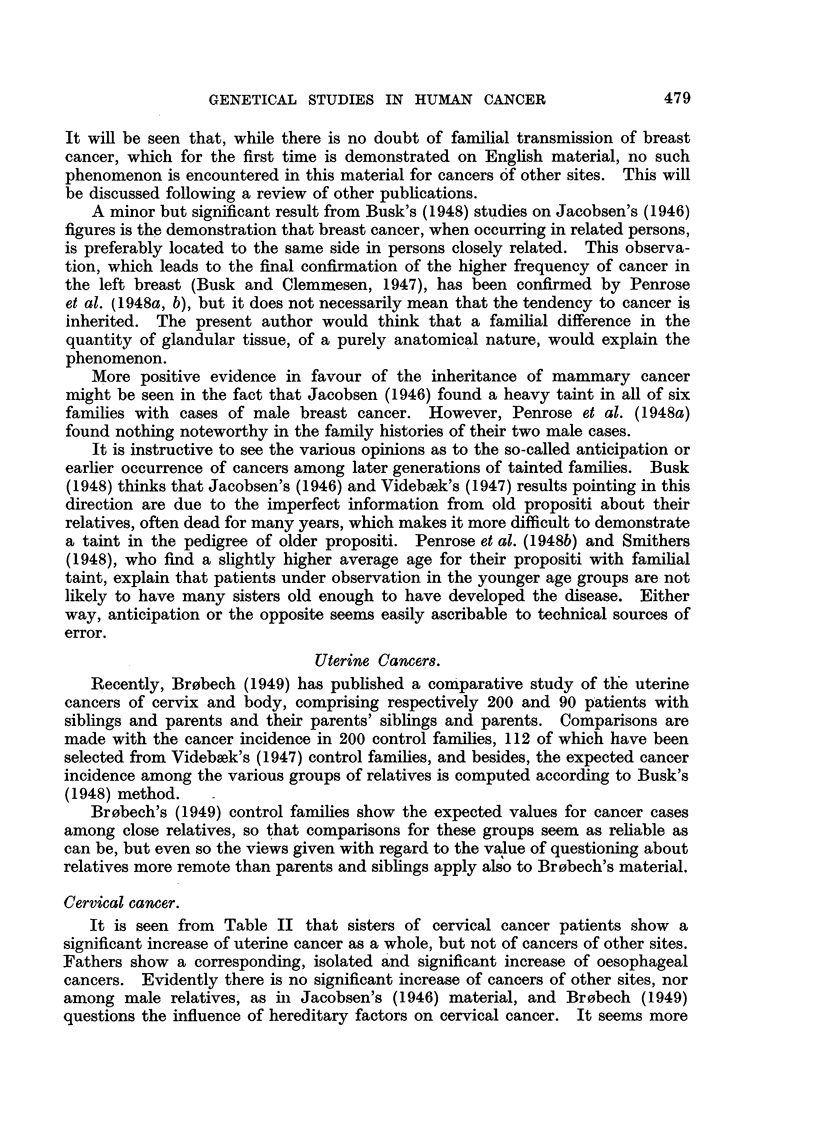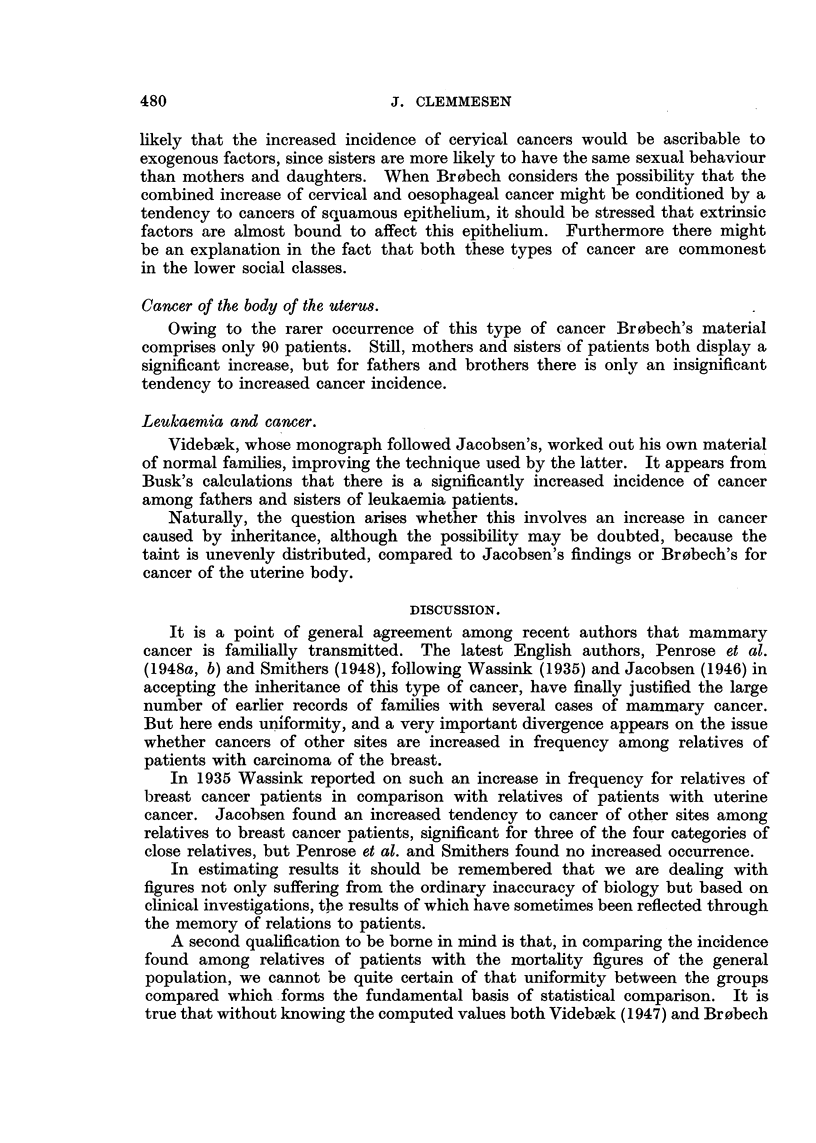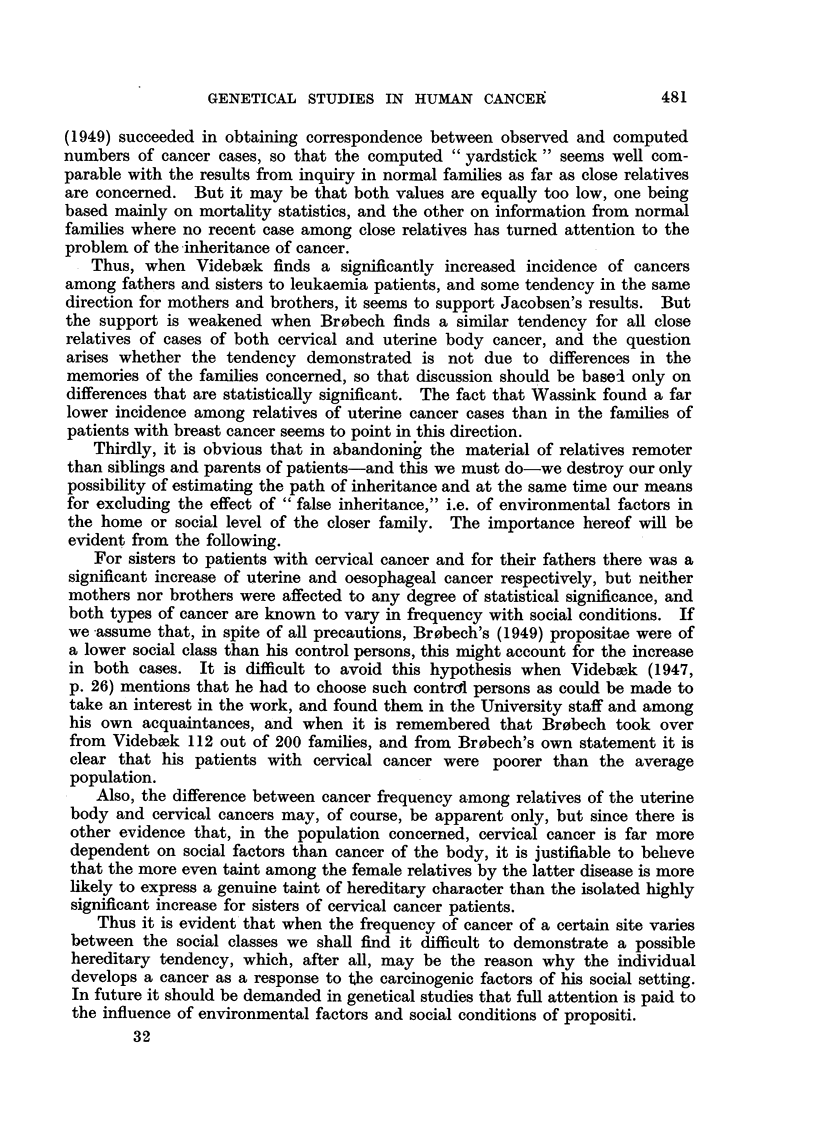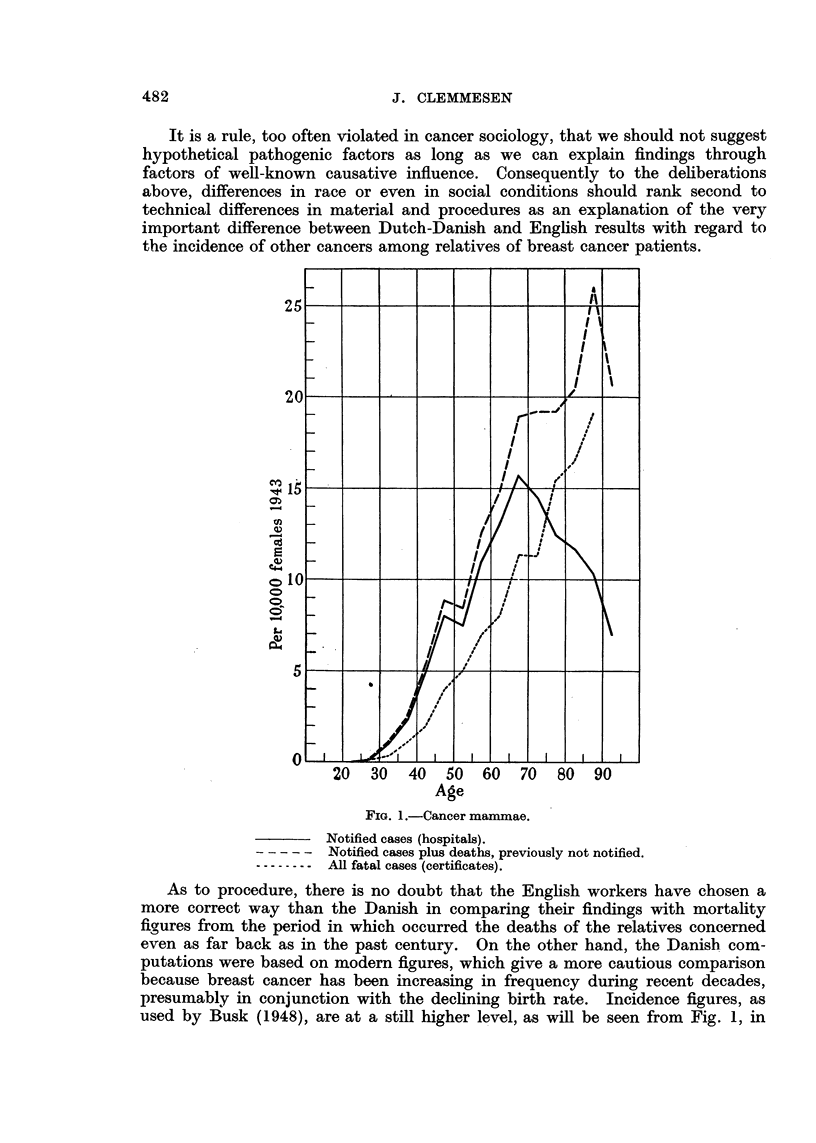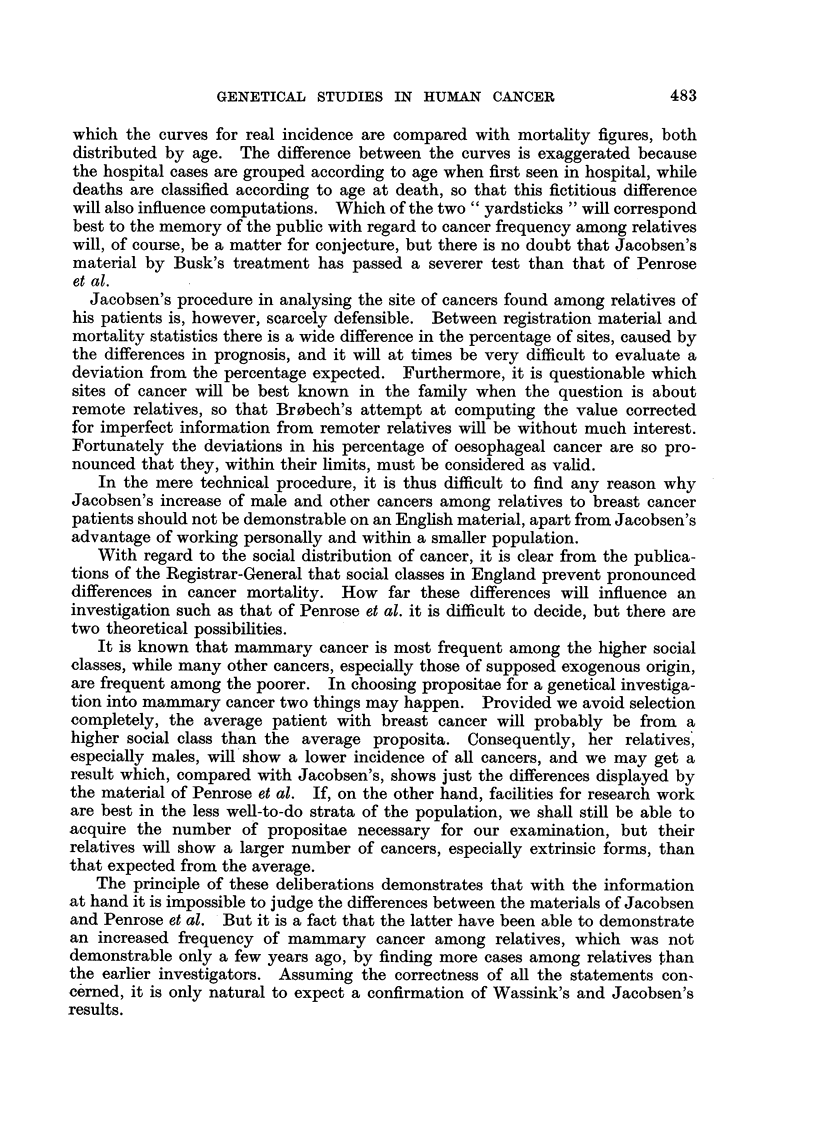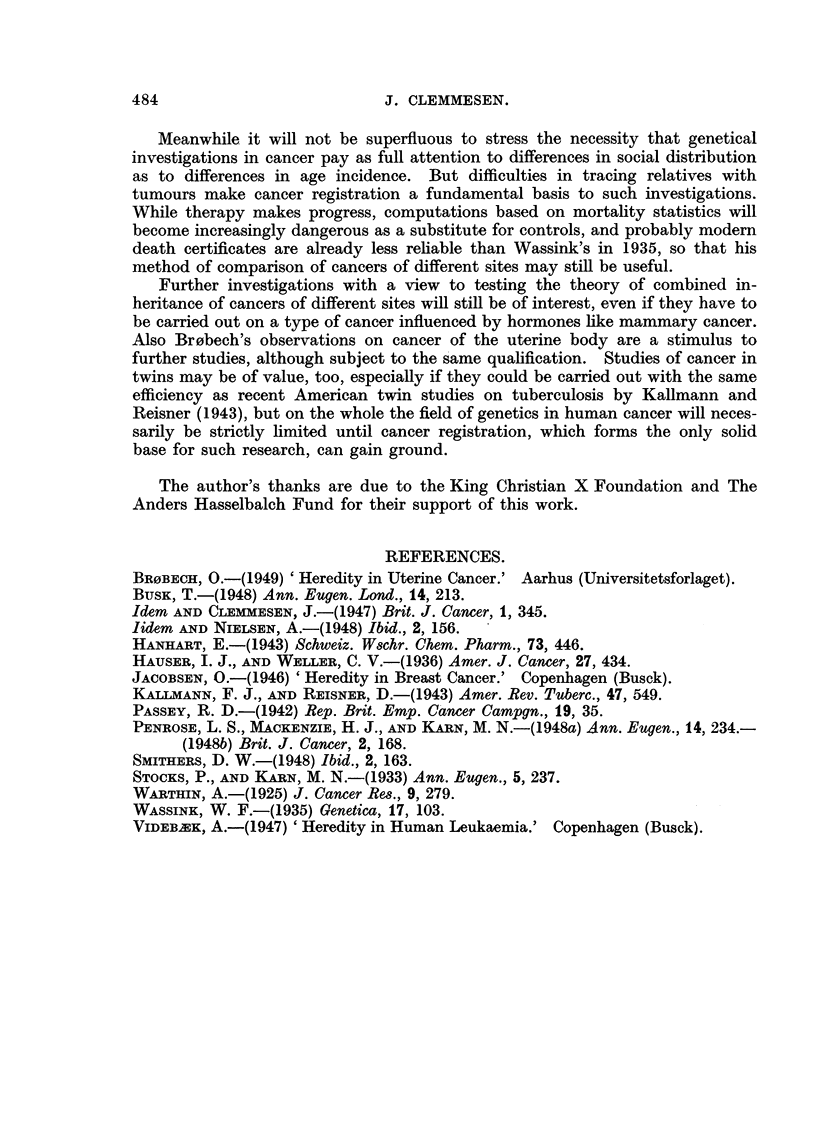# The Status of Genetical Studies in Human Cancer

**DOI:** 10.1038/bjc.1949.50

**Published:** 1949-12

**Authors:** J. Clemmesen


### THE STATUS OF GENETICAL STUDIES IN HUMAN CANCER.

J. CLEMMESEN.

*From the Danish Cancer Registry under the National Anti-Cancer League, Copenhagen.*

Received for publication September 23, 1949.

A PROBLEM fundamental in research on human cancer is the question whether there are causative factors common to cancers of different sites, and, if so, to what extent. It would speak in favour of the existence of such factors if it were possible to demonstrate an increased tendency to cancer among male relatives of women with cancers of specifically female type.

In " Family G " described by Warthin (1925) 15 out of 23 cancers in women were adenocarcinomata of the endometrium, while all the 20 cancers in men were localized to the stomach or intestine (Hauser and Weller, 1936). Wassink (1935) has also produced results pointing in this direction, but the solution of the problem mentioned will demand further evidence.

Publications in recent years on the heredity of human cancer display some divergence of results, which may appear bewildering to the student of cancer interested in the problem but unfamiliar with genetics. Furthermore, it is sometimes forgotten that the difficulties encountered in this field are not only genetical but also clinical and sociological, so that conclusions reached by geneticists in apparent safety may become endangered through progress in clinical technique and sociology. Even quite extensive work has sometimes resulted only in this knowledge, and consequently in the obligation to pass it on. For these various reasons a synthesis of some recent publications on the heredity of human cancers will be attempted here.

In 1942 the University Institute for Human Genetics and the Danish Cancer Registry, both in Copenhagen, entered into collaboration to examine the extent to which some types of human cancer were inherited, thus exploiting the unusual opportunity afforded by a small population under registration for the disease in question, uniform and well-suited for the purpose, both mentally, socially and racially, all members having equal access to first-class medical facilities.

The publication of the results from these studies was followed by contributions from other authors, in some cases of a critical character, and the whole complex of monographs and articles will briefly be reviewed here.

The choice of cancer types for the investigation will serve to illustrate the character of the task, and the difficulties in evaluating results. It is vital that the cancer chosen is accessible to the establishment of a diagnosis with some certainty, not so much with regard to propositi themselves as with a view to their relatives, the diagnoses of whose cancers have often been made years ago and under less favourable conditions—a factor far too often overlooked in studies of this kind. Consequently, the abdomen and the lung were out of the question as sites for the investigations planned.

Prognosis is also of importance. Cancer of the skin is unsuited, because the easy treatment and favourable prognosis leave the patient and his relatives ignorant of the severity of the affection, and thus forgetful about its existence. The high age of onset of this disease is also a disadvantage, because relatives of patients being older on the average will have had their diagnoses made some decades earlier, and thus under circumstances different from the present.

An unfavourable prognosis may, on the other hand, fatally diminish the number of patients available for an interview. Oesophageal cancer presents this difficulty, combined with high average age and often with poor social conditions. Dr. Mogensen, otologist, made a heroic effort in collecting 75 families in spite of all difficulties, but met with the ultimate obstacle that heavy drinking is very common, not only among the later patients, but also among their relatives. So even a pronounced familial occurrence of oesophageal cancer might be due to the inheritance of the mental qualities of habitual drinking, which habit, directly or indirectly, is associated with the development of oesophageal cancers.

Rectal cancer was deemed unsuited for investigation because of the difficulty in excluding the influence of inheritable intestinal polyposis on the development of cancers in this site.

Thus mammary cancer (Jacobsen, 1946), leukaemia (Videbæk, 1947) and the uterine cancers of cervix and corpus (Brøbech, 1949) were chosen for the investigation. It was an advantage that all these affections have an early age of onset, but it had to be realized that the differential diagnosis of the latter three diseases will often be less firmly established for relatives than for propositi themselves.

The technique of the three investigators was identical in principle, although some improvements were introduced in the course of their studies. About 200 patients were, as a rule, interviewed by the investigator in person about their relatives, their data and diseases. Since birth dates are always known in the Danish population, it was possible to see to it that the age distribution of propositi corresponded closely to the distribution of all cases in the country. A corresponding number of carefully selected normal persons, as far as possible of corresponding age, etc., were likewise questioned. All diagnoses of cancer and of many of the remaining fatal cases were, as far as possible, confirmed through death certificates or case records from hospitals. This applies, for instance, to all cases of cancer among close relatives of Jacobsen's breast cancer patients. Detailed pedigrees are given for all patients with mammary cancer and leukaemia in Jacobsen's (1946) and Videbæk's (1947) monographs, so that every case can be easily checked.

With some aid from Jacobsen these possibilities for further studies have been exploited for a critical review by Busk (1948), and Busk, Clemmesen and Nielsen (1948). Busk's (1948) computations are based on the idea that control persons will never take quite the same interest in cancer cases among their relatives as do patients and their families, to whom the question about the inheritance of cancer has become topical. However, mortality figures will be serviceable as a " yardstick " comparable to the memory of relatives, which will most often be limited to fatal cases, so that the more correct figures from a cancer registry will be less useful. Exceptionally, Busk has replaced mortality figures for mammary cancer by the Cancer Registry's figures for incidence, because of the assumption that this affection would be regarded by Danish laymen as a dangerous but not incurable affection to be remembered in the family even if cured, while natural shyness would prevent uterine cancers from being discussed and remembered in the same way, so that mortality figures suffice for the latter as for the remaining sites.

On this basis it was possible to compute the number of cancers expected in each group of relatives with full allowance for the variation in age distribution. For relatives to control persons, Busk (1948) computed that Jacobsen (1946) had succeeded in finding only 81, or 29 per cent, out of 278 cancer cases expected among siblings and parents, and parents' siblings and parents. Videbæk (1947), benefiting from Jacobsen's experience, found 218 out of 300, or 73 per cent, presumably because he systematically interviewed more than one person in each family. For parents and siblings, however, Videbæk's figures for normal families show good correspondence with the expected values, and consequently we shall limit ourselves to such conclusions as can be drawn from the findings among siblings and parents of patients, termed " close relatives," since there seems to be justification for accepting the " yardstick " as practically valid for these categories.

*Mammary Cancer.*

From Jacobsen's (1946) review of previous studies it appears that results interpreted as positive with regard to inheritance of mammary cancer date as far back as one century, but even in the last two decades authors of reputation (Stocks and Karn, 1933 ; Passey, 1942 ; Hanhart, 1943) find no evidence that a history of cancer in relatives increases the risk of cancer to any measurable extent.

An important reason for doubting the positive results was the discussion about the validity of control materials as an expression of the full number of cancers occurring among relatives of normal persons. However, by Busk's (1948) computations on Jacobsen's (1946) results it has been demonstrated that close relatives of patients with mammary cancer show a significantly increased number of cancer cases. This result is beyond doubt, since all the cases among close relatives have been confirmed through hospital case records or death certificates, and since the control figures for breast cancer itself have been computed from real incidence figures, which, as will be seen later, make a severer test that mortality figures.

It will appear from Table I that there is not only an increase in the incidence of mammary cancer among the female relatives of the patients, but also a significant increase in the incidence of cancers at other sites among mothers, while sisters show a tendency in the same direction. Male relatives also display a significant increase of cancers.

Thus Jacobsen's (1946) two main theses, (1) on the increased incidence of mammary cancer, and (2) on the increased incidence of other cancers among the relatives of breast cancer patients, have been borne out by his first critics, but further evidence on the subject has been brought forward by Smithers (1948), and by Penrose, Mackenzie and Karn (1948*b*).

Smithers (1948) reported on 459 family histories of patients suffering from breast cancer. The histories had been taken at the time of the patients' first visit to hospital, and had at the time of publication not yet been supplemented by later questions to propositae or relatives. Apparently the material was not collected by the same investigator, but Smithers, who does not overestimate the value of his material, rightly thinks it worthy of notice that the figures show an unduly high incidence of cancer of the breast, and *not of other cancers.*

Some part of Smithers' (1948) material is included in the figures given by Penrose *et al.* (1948*a*), of which those for close relatives are quoted in Table I.

TABLE I.—*Incidence of Cancer Cases in Relatives of Patients and Control Persons.*

| | Patients' relatives. Breast. Observed. | Patients' relatives. Breast. Expected. | Patients' relatives. Other sites. Observed. | Patients' relatives. Other sites. Expected. | | Control families. Breast. Observed. | Control families. Breast. Expected. | Control families. Other sites. Observed. | Control families. Other sites. Expected. |
|---|---|---|---|---|---|---|---|---|---|
| Jacobsen (1946); Busk, (1948): | | | | | | | | | |
| 200 Mothers | 21‡ | 7 | 34* | 22 | 200 Mothers | 2 | 4 | 17 | 23 |
| 381 Sisters | 13‡ | 5 | 17 | 11 | 433 Sisters | 2 | 2 | 6 | 10 |
| 199 Fathers | 1 | .. | 39‡ | 23 | 199 Fathers | .. | .. | 19 | 22 |
| 377 Brothers | .. | .. | 16* | 8 | 389 Brothers | .. | .. | 4 | 10 |
| Penrose, Mackenzie and Karn (1948*a*): | | | | | | | | | |
| 406 Mothers | 25 | 11·12 | 51 | 49·23 | | | | | |
| 307 Sisters | 23 | 6·97 | 19 | 25·23 | | | | | |
| 420 Fathers | .. | 0·10 | 41 | 52·32 | | | | | |
| 455 Brothers | .. | .. | 29 | 28·14 | | | | | |

* Signifies that the probability of the difference found, under the assumption that the true incidence was the same for the two materials compared, amounts to between 5 and 1 per cent.

‡ Signifies correspondingly a probability below 1 per thousand.

TABLE II.—*Incidence of Cancer Cases in Relatives of Patients and Control Persons.*

| Brøbech (1949): | Patients' relatives. Uterus. Observed. | Patients' relatives. Uterus. Expected. | Patients' relatives. Other sites. Observed. | Patients' relatives. Other sites. Expected. | | Control families. Uterus. Observed. | Control families. Uterus. Expected. | Control families. Other sites. Observed. | Control families. Other sites. Expected. |
|---|---|---|---|---|---|---|---|---|---|
| *Carcinoma of the cervix.* | | | | | | | | | |
| 200 Mothers | 6 | 3·0 | 22 | 20·3 | 200 Mothers | 6 | 3·1 | 19 | 21·1 |
| 488 Sisters | 10† | 2·2 | 12 | 7·7 | 449 Sisters | 1 | 2·1 | 8 | 7·6 |
| 200 Fathers | .. | .. | 26 | 19·7 | 200 Fathers | .. | .. | 22 | 19·5 |
| 495 Brothers | .. | .. | 9 | 8·2 | 423 Brothers | .. | .. | 4 | 6·6 |
| *Carcinoma of the body of the uterus.* | | | | | | | | | |
| 90 Mothers | 6* | 1·7 | 16 | 13·3 | 90 Mothers | 3 | 1·7 | 12 | 14·0 |
| 217 Sisters | 6* | 1·8 | 11 | 7·7 | 233 Sisters | 1 | 1·8 | 10 | 8·0 |
| 90 Fathers | .. | .. | 18 | 13·0 | 90 Fathers | .. | .. | 10 | 11·9 |
| 184 Brothers | .. | .. | 8 | 6·5 | 212 Brothers | .. | .. | 6 | 6·0 |

Videbæk (1947); Busk (1948):

*Leukaemia.*

| | Patients' relatives. Cancer all sites. Observed. | Patients' relatives. Cancer all sites. Expected. | | Control families. Cancer all sites. Observed. | Control families. Cancer all sites. Expected. |
|---|---|---|---|---|---|
| 208 Fathers | 31* | 20 | 200 Fathers | 18 | 19 |
| 457 Brothers | 18 | 15 | 393 Brothers | 9 | 14 |
| 209 Mothers | 28 | 24 | 200 Mothers | 21 | 22 |
| 447 Sisters | 32† | 18 | 409 Sisters | 15 | 17 |

* As in Table I.
† Signifies a probability between 1 and 0·1 per cent.

It will be seen that, while there is no doubt of familial transmission of breast cancer, which for the first time is demonstrated on English material, no such phenomenon is encountered in this material for cancers of other sites. This will be discussed following a review of other publications.

A minor but significant result from Busk's (1948) studies on Jacobsen's (1946) figures is the demonstration that breast cancer, when occurring in related persons, is preferably located to the same side in persons closely related. This observation, which leads to the final confirmation of the higher frequency of cancer in the left breast (Busk and Clemmesen, 1947), has been confirmed by Penrose *et al.* (1948*a*, *b*), but it does not necessarily mean that the tendency to cancer is inherited. The present author would think that a familial difference in the quantity of glandular tissue, of a purely anatomical nature, would explain the phenomenon.

More positive evidence in favour of the inheritance of mammary cancer might be seen in the fact that Jacobsen (1946) found a heavy taint in all of six families with cases of male breast cancer. However, Penrose *et al.* (1948*a*) found nothing noteworthy in the family histories of their two male cases.

It is instructive to see the various opinions as to the so-called anticipation or earlier occurrence of cancers among later generations of tainted families. Busk (1948) thinks that Jacobsen's (1946) and Videbæk's (1947) results pointing in this direction are due to the imperfect information from old propositi about their relatives, often dead for many years, which makes it more difficult to demonstrate a taint in the pedigree of older propositi. Penrose *et al.* (1948*b*) and Smithers (1948), who find a slightly higher average age for their propositi with familial taint, explain that patients under observation in the younger age groups are not likely to have many sisters old enough to have developed the disease. Either way, anticipation or the opposite seems easily ascribable to technical sources of error.

### *Uterine Cancers.*

Recently, Brøbech (1949) has published a comparative study of the uterine cancers of cervix and body, comprising respectively 200 and 90 patients with siblings and parents and their parents' siblings and parents. Comparisons are made with the cancer incidence in 200 control families, 112 of which have been selected from Videbæk's (1947) control families, and besides, the expected cancer incidence among the various groups of relatives is computed according to Busk's (1948) method.

Brøbech's (1949) control families show the expected values for cancer cases among close relatives, so that comparisons for these groups seem as reliable as can be, but even so the views given with regard to the value of questioning about relatives more remote than parents and siblings apply also to Brøbech's material.

#### *Cervical cancer.*

It is seen from Table II that sisters of cervical cancer patients show a significant increase of uterine cancer as a whole, but not of cancers of other sites. Fathers show a corresponding, isolated and significant increase of oesophageal cancers. Evidently there is no significant increase of cancers of other sites, nor among male relatives, as in Jacobsen's (1946) material, and Brøbech (1949) questions the influence of hereditary factors on cervical cancer. It seems more likely that the increased incidence of cervical cancers would be ascribable to exogenous factors, since sisters are more likely to have the same sexual behaviour than mothers and daughters. When Brøbech considers the possibility that the combined increase of cervical and oesophageal cancer might be conditioned by a tendency to cancers of squamous epithelium, it should be stressed that extrinsic factors are almost bound to affect this epithelium. Furthermore there might be an explanation in the fact that both these types of cancer are commonest in the lower social classes.

*Cancer of the body of the uterus.*

Owing to the rarer occurrence of this type of cancer Brøbech's material comprises only 90 patients. Still, mothers and sisters of patients both display a significant increase, but for fathers and brothers there is only an insignificant tendency to increased cancer incidence.

*Leukaemia and cancer.*

Videbæk, whose monograph followed Jacobsen's, worked out his own material of normal families, improving the technique used by the latter. It appears from Busk's calculations that there is a significantly increased incidence of cancer among fathers and sisters of leukaemia patients.

Naturally, the question arises whether this involves an increase in cancer caused by inheritance, although the possibility may be doubted, because the taint is unevenly distributed, compared to Jacobsen's findings or Brøbech's for cancer of the uterine body.

### DISCUSSION.

It is a point of general agreement among recent authors that mammary cancer is familially transmitted. The latest English authors, Penrose *et al.* (1948*a*, *b*) and Smithers (1948), following Wassink (1935) and Jacobsen (1946) in accepting the inheritance of this type of cancer, have finally justified the large number of earlier records of families with several cases of mammary cancer. But here ends uniformity, and a very important divergence appears on the issue whether cancers of other sites are increased in frequency among relatives of patients with carcinoma of the breast.

In 1935 Wassink reported on such an increase in frequency for relatives of breast cancer patients in comparison with relatives of patients with uterine cancer. Jacobsen found an increased tendency to cancer of other sites among relatives to breast cancer patients, significant for three of the four categories of close relatives, but Penrose *et al.* and Smithers found no increased occurrence.

In estimating results it should be remembered that we are dealing with figures not only suffering from the ordinary inaccuracy of biology but based on clinical investigations, the results of which have sometimes been reflected through the memory of relations to patients.

A second qualification to be borne in mind is that, in comparing the incidence found among relatives of patients with the mortality figures of the general population, we cannot be quite certain of that uniformity between the groups compared which forms the fundamental basis of statistical comparison. It is true that without knowing the computed values both Videbæk (1947) and Brøbech

(1949) succeeded in obtaining correspondence between observed and computed numbers of cancer cases, so that the computed " yardstick " seems well comparable with the results from inquiry in normal families as far as close relatives are concerned. But it may be that both values are equally too low, one being based mainly on mortality statistics, and the other on information from normal families where no recent case among close relatives has turned attention to the problem of the inheritance of cancer.

Thus, when Videbæk finds a significantly increased incidence of cancers among fathers and sisters to leukaemia patients, and some tendency in the same direction for mothers and brothers, it seems to support Jacobsen's results. But the support is weakened when Brøbech finds a similar tendency for all close relatives of cases of both cervical and uterine body cancer, and the question arises whether the tendency demonstrated is not due to differences in the memories of the families concerned, so that discussion should be based only on differences that are statistically significant. The fact that Wassink found a far lower incidence among relatives of uterine cancer cases than in the families of patients with breast cancer seems to point in this direction.

Thirdly, it is obvious that in abandoning the material of relatives remoter than siblings and parents of patients—and this we must do—we destroy our only possibility of estimating the path of inheritance and at the same time our means for excluding the effect of " false inheritance," i.e. of environmental factors in the home or social level of the closer family. The importance hereof will be evident from the following.

For sisters to patients with cervical cancer and for their fathers there was a significant increase of uterine and oesophageal cancer respectively, but neither mothers nor brothers were affected to any degree of statistical significance, and both types of cancer are known to vary in frequency with social conditions. If we assume that, in spite of all precautions, Brøbech's (1949) propositae were of a lower social class than his control persons, this might account for the increase in both cases. It is difficult to avoid this hypothesis when Videbæk (1947, p. 26) mentions that he had to choose such control persons as could be made to take an interest in the work, and found them in the University staff and among his own acquaintances, and when it is remembered that Brøbech took over from Videbæk 112 out of 200 families, and from Brøbech's own statement it is clear that his patients with cervical cancer were poorer than the average population.

Also, the difference between cancer frequency among relatives of the uterine body and cervical cancers may, of course, be apparent only, but since there is other evidence that, in the population concerned, cervical cancer is far more dependent on social factors than cancer of the body, it is justifiable to believe that the more even taint among the female relatives by the latter disease is more likely to express a genuine taint of hereditary character than the isolated highly significant increase for sisters of cervical cancer patients.

Thus it is evident that when the frequency of cancer of a certain site varies between the social classes we shall find it difficult to demonstrate a possible hereditary tendency, which, after all, may be the reason why the individual develops a cancer as a response to the carcinogenic factors of his social setting. In future it should be demanded in genetical studies that full attention is paid to the influence of environmental factors and social conditions of propositi.

It is a rule, too often violated in cancer sociology, that we should not suggest hypothetical pathogenic factors as long as we can explain findings through factors of well-known causative influence. Consequently to the deliberations above, differences in race or even in social conditions should rank second to technical differences in material and procedures as an explanation of the very important difference between Dutch-Danish and English results with regard to the incidence of other cancers among relatives of breast cancer patients.

FIG. 1.—Cancer mammae.

———— Notified cases (hospitals).
– – – – – Notified cases plus deaths, previously not notified.
········ All fatal cases (certificates).

As to procedure, there is no doubt that the English workers have chosen a more correct way than the Danish in comparing their findings with mortality figures from the period in which occurred the deaths of the relatives concerned even as far back as in the past century. On the other hand, the Danish computations were based on modern figures, which give a more cautious comparison because breast cancer has been increasing in frequency during recent decades, presumably in conjunction with the declining birth rate. Incidence figures, as used by Busk (1948), are at a still higher level, as will be seen from Fig. 1, in which the curves for real incidence are compared with mortality figures, both distributed by age. The difference between the curves is exaggerated because the hospital cases are grouped according to age when first seen in hospital, while deaths are classified according to age at death, so that this fictitious difference will also influence computations. Which of the two " yardsticks " will correspond best to the memory of the public with regard to cancer frequency among relatives will, of course, be a matter for conjecture, but there is no doubt that Jacobsen's material by Busk's treatment has passed a severer test than that of Penrose *et al.*

Jacobsen's procedure in analysing the site of cancers found among relatives of his patients is, however, scarcely defensible. Between registration material and mortality statistics there is a wide difference in the percentage of sites, caused by the differences in prognosis, and it will at times be very difficult to evaluate a deviation from the percentage expected. Furthermore, it is questionable which sites of cancer will be best known in the family when the question is about remote relatives, so that Brøbech's attempt at computing the value corrected for imperfect information from remoter relatives will be without much interest. Fortunately the deviations in his percentage of oesophageal cancer are so pronounced that they, within their limits, must be considered as valid.

In the mere technical procedure, it is thus difficult to find any reason why Jacobsen's increase of male and other cancers among relatives to breast cancer patients should not be demonstrable on an English material, apart from Jacobsen's advantage of working personally and within a smaller population.

With regard to the social distribution of cancer, it is clear from the publications of the Registrar-General that social classes in England prevent pronounced differences in cancer mortality. How far these differences will influence an investigation such as that of Penrose *et al.* it is difficult to decide, but there are two theoretical possibilities.

It is known that mammary cancer is most frequent among the higher social classes, while many other cancers, especially those of supposed exogenous origin, are frequent among the poorer. In choosing propositae for a genetical investigation into mammary cancer two things may happen. Provided we avoid selection completely, the average patient with breast cancer will probably be from a higher social class than the average proposita. Consequently, her relatives, especially males, will show a lower incidence of all cancers, and we may get a result which, compared with Jacobsen's, shows just the differences displayed by the material of Penrose *et al.* If, on the other hand, facilities for research work are best in the less well-to-do strata of the population, we shall still be able to acquire the number of propositae necessary for our examination, but their relatives will show a larger number of cancers, especially extrinsic forms, than that expected from the average.

The principle of these deliberations demonstrates that with the information at hand it is impossible to judge the differences between the materials of Jacobsen and Penrose *et al.* But it is a fact that the latter have been able to demonstrate an increased frequency of mammary cancer among relatives, which was not demonstrable only a few years ago, by finding more cases among relatives than the earlier investigators. Assuming the correctness of all the statements concerned, it is only natural to expect a confirmation of Wassink's and Jacobsen's results.

Meanwhile it will not be superfluous to stress the necessity that genetical investigations in cancer pay as full attention to differences in social distribution as to differences in age incidence. But difficulties in tracing relatives with tumours make cancer registration a fundamental basis to such investigations. While therapy makes progress, computations based on mortality statistics will become increasingly dangerous as a substitute for controls, and probably modern death certificates are already less reliable than Wassink's in 1935, so that his method of comparison of cancers of different sites may still be useful.

Further investigations with a view to testing the theory of combined inheritance of cancers of different sites will still be of interest, even if they have to be carried out on a type of cancer influenced by hormones like mammary cancer. Also Brøbech's observations on cancer of the uterine body are a stimulus to further studies, although subject to the same qualification. Studies of cancer in twins may be of value, too, especially if they could be carried out with the same efficiency as recent American twin studies on tuberculosis by Kallmann and Reisner (1943), but on the whole the field of genetics in human cancer will necessarily be strictly limited until cancer registration, which forms the only solid base for such research, can gain ground.

The author's thanks are due to the King Christian X Foundation and The Anders Hasselbalch Fund for their support of this work.

### REFERENCES.

Brøbech, O.—(1949) 'Heredity in Uterine Cancer.' Aarhus (Universitetsforlaget).
Busk, T.—(1948) *Ann. Eugen. Lond.*, **14**, 213.
*Idem* and Clemmesen, J.—(1947) *Brit. J. Cancer*, **1**, 345.
*Iidem* and Nielsen, A.—(1948) *Ibid.*, **2**, 156.
Hanhart, E.—(1943) *Schweiz. Wschr. Chem. Pharm.*, **73**, 446.
Hauser, I. J., and Weller, C. V.—(1936) *Amer. J. Cancer*, **27**, 434.
Jacobsen, O.—(1946) 'Heredity in Breast Cancer.' Copenhagen (Busck).
Kallmann, F. J., and Reisner, D.—(1943) *Amer. Rev. Tuberc.*, **47**, 549.
Passey, R. D.—(1942) *Rep. Brit. Emp. Cancer Campgn.*, **19**, 35.
Penrose, L. S., Mackenzie, H. J., and Karn, M. N.—(1948*a*) *Ann. Eugen.*, **14**, 234.—(1948*b*) *Brit. J. Cancer*, **2**, 168.
Smithers, D. W.—(1948) *Ibid.*, **2**, 163.
Stocks, P., and Karn, M. N.—(1933) *Ann. Eugen.*, **5**, 237.
Warthin, A.—(1925) *J. Cancer Res.*, **9**, 279.
Wassink, W. F.—(1935) *Genetica*, **17**, 103.
Videbæk, A.—(1947) 'Heredity in Human Leukaemia.' Copenhagen (Busck).